# The Effect of Quintuply-Fortified Salt on the Gut Microbiome of Young Children 1–5 y of Age in Punjab, India; A Substudy of a Randomized, Community-Based Trial

**DOI:** 10.1016/j.cdnut.2025.107580

**Published:** 2025-10-23

**Authors:** Lauren Thompson, Yvonne E Goh, Manu Jamwal, Bidhi L Singh, Gurjinder Kaur Brar, Charles D Arnold, Jamie Westcott, Julie M Long, Nancy F Krebs, Angela Zivkovic, Reena Das, Mona Duggal, Christine M McDonald

**Affiliations:** 1 Institute for Global Nutrition, University of California, Davis, CA, United States; 2 Departments of Pediatrics and Epidemiology and Biostatistics, University of California, San Francisco, CA, United States; 3 International Zinc Nutrition Consultative Group, San Francisco, CA, United States; 4 Postgraduate Institute of Medical Education and Research, Chandigarh, India; 5 Department of Pediatrics, Section of Nutrition, University of Colorado Anschutz Medical Campus, Aurora, CO, United States; 6 Department of Nutrition, University of California, Davis, CA, United States

**Keywords:** large-scale food fortification, micronutrients, microbiome, iron, fortification, condiment fortification, salt fortification, children

## Abstract

**Background:**

Young children in India often face multiple micronutrient deficiencies, yet interventions such as micronutrient powders have raised concerns about potential adverse effects on the gut microbiome. Large-scale food fortification is an effective strategy to improve micronutrient intake; however, its impact on the gut microbiome of children remains unclear.

**Objectives:**

To determine whether intake of quintuply-fortified salt (QFS) for 12 mo adversely affects gut microbiome composition in children aged 1–5 y.

**Methods:**

In a double-blind, randomized, controlled trial in Punjab, India, children received: *1*) QFS with iron as encapsulated ferrous fumarate [eFF], zinc, vitamin B12, folic acid, and iodine (eFF-QFS); *2*) QFS with the same micronutrients, but iron as encapsulated ferric pyrophosphate [eFePP] plus ethylenediaminetetraacetic acid (eFePP-QFS); or *3*) standard iodized salt for 12 mo. Stool samples were collected from 125 children (eFF-QFS, *n* = 43; eFePP-QFS, *n* = 45; iodized salt, *n*= 37) at baseline and 12 mo and analyzed via 16S rRNA gene sequencing. Changes in alpha diversity (Shannon, abundance-based estimator index) between groups were assessed with linear mixed models, beta diversity (Bray-Curtis dissimilarity) with linear regression and permutational multivariate analysis of variance, and relative abundance of *Enterobacteriaceae*, *Lactobacillus*, *Bifidobacterium*, *Bacteroides*, *Prevotella*, or *Escherichia-Shigella* with zero-inflated negative binomial mixed models.

**Results:**

Average discretionary salt utilization was estimated to be 3.5 g/child equivalent/d across groups. Abundance-based estimator index was higher in the iodized salt arm compared with eFePP-QFS, but similar to eFF-QFS. Permutational multivariate analysis of variance revealed no overall group differences; however, pairwise Bray-Curtis distances from baseline were modestly greater in eFF-QFS compared with the other groups. No significant changes in relative abundance were identified.

**Conclusions:**

After 12 mo, QFS resulted no major changes in abundance of key taxa and minimal, inconsistent shifts in certain diversity metrics and relative to the iodized salt control, suggesting no adverse effects on microbiome composition among young children in this setting. Additional studies in settings with improved iron status are needed.

This trial was registered at clinicaltrials.gov as NCT05166980 and at Clinical Trials Registry–India as CTRI/2022/02/040333.

## Introduction

Micronutrient deficiencies are widespread among young children in India, particularly in the northern state of Punjab. According to the most recent Comprehensive National Nutrition Survey (2016–2018) [1], the national prevalence of iron deficiency among preschool age children under 5 y of age was 32%. In Punjab, the prevalence of iron deficiency was even higher, at 67.2% [1]. The national prevalence of zinc deficiency was 19% in that same age group, rising to over 50% in Punjab. Punjab also demonstrates disproportionately high prevalences of folate and vitamin B12 deficiencies among young children [1]. These combined deficits can result in severe health, growth, and developmental consequences [2], emphasizing the need for multiple-micronutrient interventions in this region.

Several strategies have been recommended to address micronutrient deficiencies among individuals and populations at risk for deficiencies. Supplementation and home fortification, including provision of iron supplements and point-of-use micronutrient powders (MNPs), are targeted strategies to close dietary micronutrient gaps among children [3]. Large-scale food fortification (LSFF), or the addition of one or more vitamins and minerals to commonly consumed staple foods or condiments, is another strategy which is designed to improve micronutrient intake at the population level. Both approaches have consistently proven to improve biomarkers of micronutrient status [4,5].

However, there is concern that the level of iron provided in many micronutrient interventions could inadvertently harm gut health. Because of the low fractional absorption (<20%) of iron from iron fortificants and supplements, the majority of ingested iron remains unabsorbed in the colon [6]. For many gram-negative bacteria, iron is essential for colonization and virulence, and excess colonic iron may produce an environment conducive to pathogenic growth [7,8]. This can result in adverse shifts in the gut microbiome, promote intestinal inflammation, and increase the risk of diarrhea [6]. Specifically, iron-containing MNPs have come under scrutiny, with several studies linking their use to increased diarrhea incidence among young children [6,[9], [10], [11], [12], [13]]. Some studies have reported increases in the relative abundance of *Enterobacteriaceae* [12,14], a bacterial family that includes genus groups such as *Escherichia-Shigella*, *Campylobacter*, and *Clostridium*; all of which have also been shown to increase in response to MNPs [11,12]. These genus groups contain pathogenic species such as pathogenic *Escherichia coli*, *Campylobacter jejuni*, and *Clostridium difficile*, which are commonly associated with diarrheal diseases. In particular, Jaeggi et al. [10] observed an increase in pathogenic strains of *E. coli* after MNP supplementation. These shifts have also been accompanied by declines in beneficial bacteria such as *Bifidobacterium* and *Lactobacillus* [12]. This is especially important in children, as microbiome alterations have been associated with poor growth [15,16], and diarrhea carries an elevated risk of mortality [[17], [18], [19]].

Micronutrient interventions that deliver a lower effective level of iron may help mitigate the potential negative effects that have been associated with higher iron doses and maximize efficiency in preventing micronutrient deficiencies. LSFF is a promising strategy, offering a cost-effective, sustainable approach to increase micronutrient intake of the entire population that requires no behavior change and leverages existing delivery platforms. By delivering smaller amounts of iron over an extended period of time, LSFF could help minimize potential adverse effects on the gut. However, the impact of LSFF on the gut microbiome is not well understood, underscoring the need for additional research.

To address this evidence gap, we aim to leverage data from a double-blind randomized, controlled, community-based trial in Punjab, India, that compared quintuply-fortified salt (QFS) with iron as either encapsulated ferrous fumarate (eFF) or encapsulated ferric pyrophosphate (eFePP) plus EDTA, in addition to zinc, folic acid, vitamin B12, and iodine to an iodized salt control group for the improvement of micronutrient status among women and their children. This study used 16S rRNA gene sequencing to identify changes in the gut microbiome of children 1–5 y of age after exposure to QFS for 12 mo. To assess the safety profile of QFS, we evaluated for adverse shifts in overall bacterial diversity (alpha and beta diversity) and for changes in key iron-responsive taxa, including increases in *Escherichia-Shigella* and *Enterobacteriaceae* alongside decreases in *Bifidobacterium*, *Lactobacillus*, and *Bacteroides*.

## Methods

### Study design

This study was designed as a substudy of a double-blind, randomized, controlled, community-based, trial that took place in the Mohali District of Punjab, India. The full details of the parent study design can be found in the study protocol [20]. Briefly, nonpregnant women of reproductive age were recruited to participate in the study. One of the women’s children 12–59 mo of age was also eligible to participate in the study. If a woman had >1 children 12–59 mo of age, only 1 of the children was randomly selected to participate in the study. Eligible women, children, and their households were randomly assigned to 1 of 3 study groups *1*) QFS with iron in the form of eFF, zinc as zinc oxide, vitamin B12, folic acid, and iodine as potassium iodate (eFF-QFS); *2*) QFS with the same 5 micronutrients, but with iron in the form of eFePP plus EDTA (eFePP-QFS); *3*) iodized salt. Fortification levels for eFF-QFS and eFePP-QFS per gram of salt were: 1.3 mg iron; 1.4 mg zinc; 0.6 μg vitamin B12; 52 μg folic acid, and 30 μg iodine. Formative research among nonpregnant women of reproductive age in the study area found that mean discretionary salt intake was 4.6 g [21]. The fortification levels were set to maximize reductions in the prevalence of inadequate micronutrient intake while ensuring that excessive intake among women did not exceed 5% for each micronutrient, in accordance with WHO recommendations. Although dietary intake data were not available for children during the formative research stage, we assumed that discretionary salt intake is proportional to energy intake, with the average energy intake of primary school children (PSC) being ∼50% of that of women of reproductive age. On the basis of this, the expected mean discretionary salt intake of PSC was estimated to be ∼2.3 g/d. The primary outcomes of the main trial included impacts on biomarkers of iron, zinc, vitamin B12, folate, and iodine after exposure to the study salt for 12 mo.

To be eligible participate in the parent study and the microbiome substudy, children must have met the following criteria: *1*) 12–59 mo of age; *2*) child’s mother or primary caregiver has been enrolled into the trial; *3*) not severely anemic (defined as a hemoglobin concentration <7.0 g/dL, as measured by fingerprick blood sample and HemoCue 301+); *4*) not severely acutely malnourished (defined according to a weight-for-length/height Z-score <−3 or mid-upper arm circumference <115 mm); *5*) no serious health problems that interfere with the child’s eating practices. Written and oral informed consent was obtained from the child’s mother before participation in the study. The trial protocol was approved by the Institutional Review Board of the University of California San Francisco, the Institutional Ethics Committee of the Post Graduate Institute of Medical Education and Research (PGIMER), and India’s Health Ministry’s Screening Committee. The study was registered at clinicaltrials.gov (NCT05166980) and in the Clinical Trials Registry-India (CTRI/2022/02/040333).

After confirmation of eligibility and provision of consent, study staff collected data sociodemographic status from the child’s mother, and children were subsequently scheduled for biochemical and anthropometric assessments in the next 2–3 d. If baseline data collection was completed and the blood draw was successful, women and children were randomly assigned to receive FePP-QFS, eFF-QFS, or iodized salt.

Randomization of women and their children to 1 of the 3 study groups was implemented via a computer-generated block-randomized assignment. Sealed opaque envelopes containing the color-coded group assignment were prepared in ordered stacks and women were asked to select 1 envelope that contained a group assignment. Women were then asked to select an additional opaque envelope from a separate ordered stack that randomly divided the women and her child to the microbiome subgroup. The study team remained blinded to the group assignment until after data analysis.

After randomization, initial disbursement of the study salt was provided for the mother and child, and their household. Additional study salt was provided via monthly home visits for 12 mo. Women were instructed to use the assigned salt to fulfill the cooking and consumption needs of all household members, including children, for the duration of the study, and to maintain habitual discretionary salt consumption. Households were then visited on a monthly basis at home to provide additional disbursements of study salt, as well as to weigh and collect any unused and assess acceptability. Salt utilization was calculated from the household salt disappearance data collected at monthly visits and converted into child equivalents using household roster data and standard assumptions of weight and total energy expenditure [22,23].

Biochemical and anthropometric assessments completed at enrollment were repeated at endline (12 mo) using the same protocols described above. Laboratory assessment of micronutrient and inflammatory biomarkers [serum ferritin, hemoglobin, C-reactive protein (CRP), alpha-1-glycoprotein (AGP)] were performed using standard protocols that have been published previously [24,25]. Laboratory methods are described in further detail in the study protocol [20] and in the primary endpoints publication [26].

### Stool sample collection and sequencing

Stool samples were collected from children enrolled in the microbiome subgroup after randomization (baseline) and endline (12 mo) using stool sample collection and preservation tubes (Norgen Biotek). Women were instructed to collect stool samples from their child at home using the provided collection kit. Women were asked to collect a stool sample in the morning, and sample collection tubes were subsequently retrieved by study staff within 4 h and transported to the PGIMER central laboratory for storage at −20°C. Stool samples were then shipped on dry ice to Medgenome in Bangalore, India, for sequencing.

DNA extraction from stool samples was performed using QIAamp PowerFecal Pro DNA Kit (QIAGEN) according to the manufacturer’s protocol and quantified using Qubit DNA High sensitivity assay (Invitrogen), Qubit RNA Broad Range Assay (Invitrogen), and QIAxpert (QIAGEN) to quantify DNA and ensure no contamination from RNA. Samples were diluted with water to 5 ng and were amplified targeting the full-length 16S region (1500 bp) followed by the hypervariable regions V3-V4 (460-480bp) using 16S and V3-V4 primers, respectively, in a nested polymerase chain reaction (PCR) approach. The Eppendorf Mastercycler Nexus Gradient instrument was used to perform PCR. PCR Primer sequences, reagents, and volumes, as well as PCR cycles and temperatures are specified in the Supplemental Methods (Supplemental Tables 1–5). Positive and negative controls were included in the PCR for quality control.

All PCR products were further processed for DNA library preparation using the Twist MF Library Prep Kit for Illumina. All prepared libraries were checked for fragment distribution using the 5300 Fragment Analyzer system. Prepared libraries were sequenced using the Illumina NextSeq2000 platform. Quality checks on the raw fastq files were performed using the FASTQ toolkit to assess base quality, base composition, and guanine-cytosine (GC) content. Low-quality sequence reads were excluded from the analysis. Quantitative Insights into Microbial Ecology 2 was used stich forward and reverse primers together using the join_pairs module with default parameters to create long reads >400 base pairs. De-replication was performed using derep_fulllength in VSEARCH for the identification of unique sequences. Chimeric sequences were filtered using uchime utility from VSEARCH using a de novo approach. Sequence reads were clustered into operational taxonomic units (OTUs) using the closed_reference method under a single OTU at 99% sequence identity. Singletons were filtered out. One representative sequence from each OTU was selected and classified using the Ribosomal Database Project classifier against the SILVA-138 database at 99% similarity.

### Statistical analysis

For this substudy, a sample size of ≥50 child per intervention group was estimated to provide 80% power to detect an effect size of 0.54 SDs in each outcome (alpha and beta diversity, relative abundance of taxa) between 2 groups with a level of significance of α = 0.05, using analysis of co-variance (ANCOVA) models controlling for baseline values (assuming a weak correlation of 0.1 between baseline and endline). Accounting for an attrition rate of 25%, we aimed to enroll 185 children, or ∼62 children per study group to the microbiome subgroup.

All statistical analyses were performed using R software (version 2024.09.0 + 375). All statistical tests with a *P* value <0.05 were statistically significant. All regression models were adjusted for child age and breastfeeding [“ever breastfed” (yes/no) variable]. Additional baseline covariates were selected a priori based on biological plausibility and prior evidence linking them to gut microbiome composition [16,27,28]. Selected baseline covariates included: continuous serum AGP values, continuous serum CRP values, continuous serum ferritin values, continuous hemoglobin values, length-for-age Z-score, weight-for-age Z-score, weight-for-length Z-score, continuous Household Food Insecurity Access score [29], and household assets index measured. In all models, only those baseline covariates that showed evidence of association with the outcome at *P* value <0.1 were retained to balance model parsimony with potential increase in statistical power from explaining variation in the outcome. Log-transformations were performed on CRP, AGP, and ferritin to address skewness and linearity with the outcomes, and ferritin values were corrected for inflammation [30]. For pairwise comparisons, separate linear regression models restricted to 2 groups at a time were run**,** allowing direct assessment of differences between specific intervention groups.

Shannon Diversity and abundance-based estimator (ACE) indices were used to determine overall microbial diversity within each individual (alpha diversity) and were calculated using the vegan package in R [31]. Linear mixed models were used (lmerTest package) to estimate intervention effects on each alpha diversity metric. An (intervention group∗endline) interaction term was used to quantify the intervention effect, and participant ID was included as a random effect. Residual diagnostic plots [residual plots and quantile-quantile (QQ) plots] were inspected to assess model fit and normality of residuals.

The distribution of microbial diversity between samples (beta diversity) was evaluated using Bray-Curtis distances, which were estimated using normalized OTU counts via the vegan package in R [31]. OTU counts were normalized via cumulative sum scaling using the metagenome package in R [32]. Linear regression models were used to estimate intervention effects on Bray-Curtis pairwise dissimilarity between baseline and endline measurements within individual children. Residual diagnostic plots (residual plots and QQ plots) were inspected to assess model fit and normality of residuals. Permutational multivariate analysis of variance (PERMANOVA) was also used to estimate Bray-Curtis dissimilarity between intervention groups. The intervention effect on Bray-Curtis dissimilarity was quantified in the PERMANOVA model as (intervention group∗endline), with 999 permutations to assess statistical significance. The Bray-Curtis distance matrix was also used in principal coordinates analysis (PCoA) to visualize clustering by intervention group at baseline and endline.

Intervention effects on abundance of prespecified bacterial taxa of interest (*Enterobacteriaceae*, *Lactobacillus*, *Bacteroides*, *Bifidobacterium*, *Escherichia-Shigella*, and *Prevotella*) were estimated using zero-inflated negative binomial mixed models (glmmTMB package in R [33]). These taxa were chosen based on their abundance in this population (within the top 30 most abundant genera), and the documented interaction between iron interventions and alterations in abundance of these taxa. The zero-inflated negative binomial mixed model was chosen to accommodate the distribution of the abundance data, which are count data and were over-dispersed and zero-inflated. The intervention effect was quantified using a (intervention group∗endline) interaction term in the fixed-effects portion of the model. Total OTU counts were included as an offset term to account for varying total counts and participant ID was included as a random effect. Model fit was assessed via the DHARMa package in R [34]. Statistical tests for overdispersion were conducted using the testDispersion() function, and for zero-inflation using the testZeroInflation() function. If the zero-inflation test was nonsignificant, the zero-inflation portion of the model was removed. Lastly, an exploratory differential abundance analysis was performed on all bacterial genus groups using Analysis of Microbiomes with Bias Correction 2 [35] (ANCOMBC 2) via the phyloseq and ANCOMBC R packages. *Q* values were calculated from *P* values corrected for false discovery rate using Benjamini-Hochberg correction. If taxa were identified to be significantly different according to the ANCOMBC model (*q* value < 0.05), taxa were also assessed using zero-inflated negative binomial mixed models to incorporate additional covariate adjustment and obtain regression coefficients.

## Results

Between August 2022 and July 2023, 470 children were enrolled and randomly assigned to 1 of the 3 intervention groups. After randomization, a subsample of 171 children was randomly selected to participate in the microbiome subgroup (eFePP-QFS, *n* = 61; eFF-QFS, *n* = 56; iodized salt, *n* = 54). Seventeen children in the iodized salt subgroup, 16 children in the eFePP-QFS subgroup, and 13 children in the eFF-QFS subgroup were excluded as their household did not complete the study, the child did not provide an endline stool sample, or the stool sample was excluded due to inadequate sequencing depth. Ultimately, 125 children with paired baseline and endline samples with sufficient sequencing depth were included in the analysis (eFePP-QFS, *n* = 45; eFF-QFS, *n* = 43; iodized salt, *n* = 37). Rates of attrition were not statistically different by group. A participant flow diagram is shown in Supplemental Figure 1.

Baseline characteristics were similar across groups (Table 1). Overall, average child age was nearly 40 mo. Mean (SD) length-for-age Z-score was −0.80 (1.31) across all 3 groups, and the prevalence of stunting ranged from 7% to 16.2%. Mothers of children were well educated, with >80% of mothers completing middle school or higher. More than 95% of households were food secure. However, over one-third (42.4%) of children were anemic, 63.7% had iron deficiency, and 34.4% had iron deficiency-anemia on average across all groups. Average salt utilization was estimated to be nearly 3.5 g/child equivalent/d across all groups. Considering this level of utilization, the approximate amount of iron in the study salt was nearly 4.6 mg of iron per day. Salt utilization did not differ according to study group.

**TABLE 1:** Baseline characteristics of children randomly selected to the microbiome subgroup with sufficient read depth after sequencing (*n* = 125).

Characteristics	Mean (SD) or *n* (%)		
	eFF-QFS (*n* = 43)	FePP-QFS (*n* = 45)	Iodized salt (*n* = 37)
Age (mo)	40.0 (11.5)	42.0 (10.7)	38.5 (11.9)
Sex (female)	19 (44.2%)	24 (53.3%)	12 (32.4%)
Ever breastfed	36 (83.7%)	36 (80.0%)	29 (78.4%)
Length-for-age Z-score	−0.89 (0.9)	−0.54 (1.6)	−1.03 (1.2)
Weight-for-age Z-score	−0.57 (0.9)	−0.69 (1.2)	−0.87 (1.0)
Weight-for-length Z-score	−0.09 (1.0)	−0.55 (1.2)	−0.43 (1.1)
Stunted	3 (7.0%)	7 (15.6%)	6 (16.2%)
Wasted	1 (2.3%)	8 (17.8%)	6 (16.2%)
Underweight	1 (2.3%)	5 (11.1%)	3 (8.1%)
Maternal age (y)	30.6 (4.6)	31.0 (4.9)	29.8 (4.5)
Maternal BMI (kg/m^2^)	25.6 (4.6)	25.2 (4.5)	26.3 (3.9)
Maternal educational attainment			
None	0 (0.0%)	1 (2.3%)	0 (0.0%)
Primary	4 (9.5%)	3 (6.8%)	2 (5.6%)
Middle/secondary	31 (73.8%)	33 (75.0%)	24 (66.7%)
Diploma/post-graduate	7 (16.7%)	7 (15.9%)	10 (27.8%)
Annual household income (INR)			
<10,000	4 (9.3%)	3 (6.7%)	2 (5.4%)
10,000–12,000	9 (20.9%)	10 (22.2%)	12 (32.4%)
13,000–25,000	18 (41.9%)	20 (44.4%)	13 (35.1%)
26,000–50,000	6 (14.0%)	12 (26.7%)	8 (21.6%)
>50,000	6 (14.0%)	0 (0.0%)	2 (5.4%)
Religion			
Hindu	14 (32.6%)	12 (26.7%)	10 (27.0%)
Muslim	1 (2.3%)	3 (6.7%)	1 (2.7%)
Sikh	28 (65.1%)	30 (66.7%)	26 (70.3%)
Household is food-secure^1^	42 (97.7%)	43 (95.6%)	36 (97.3%)
Average salt utilization^2^ (g/child)	3.52 (0.99)	3.61 (0.81)	3.30 (0.83)
Anemia (hemoglobin <11 g/dL)	19 (44.2%)	15 (33.3%)	19 (51.4%)
Iron deficiency (serum ferritin^1^ <12 μg/L)	26 (61.9%)	26 (57.8%)	27 (73.0%)
Iron deficiency anemia (hemoglobin <11 g/dL and serum ferritin^3^ <12 μg/L)	16 (37.2%)	11 (24.4%)	16 (43.2%)
Inflammation			
CRP ≥5 mg/L	5 (11.9%)	5 (11.1%)	1 (2.7%)
AGP ≥1 g/L	17 (40.5%)	12 (26.7%)	14 (37.8%)

Abbreviations: AGP, alpha-1 glycoprotein; CRP, C-reactive protein; eFF-QFS, quintuply-fortified salt with iron in the form of encapsulated ferrous fumarate; FePP-QFS, quintuply-fortified salt with iron in the form of ferric pyrophosphate plus EDTA.

1 Household food security was estimated using the Household Food Insecurity Access Scale.

2 Salt utilization was calculated from the household salt disappearance data collected at monthly visits and converted into child equivalents using household roster data and standard assumptions of weight and total energy expenditure.

3 Serum ferritin values were adjusted for inflammation using the Biomarkers Reflecting Inflammation and Nutritional Determinants of Anemia regression equations.

**FIGURE 1:**
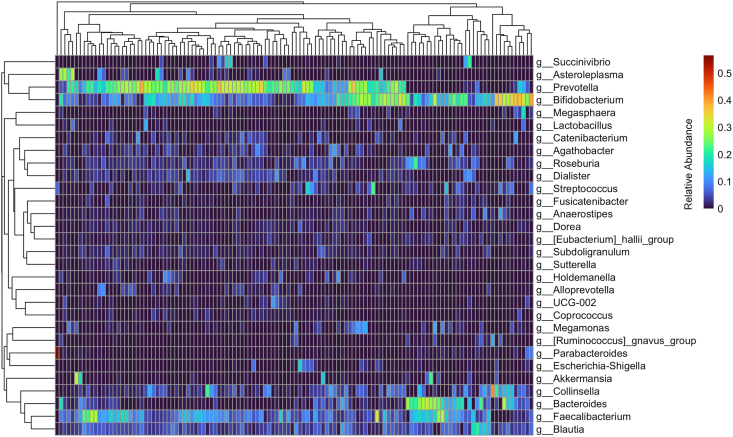
Genus-level microbiota composition at baseline among children at baseline. Heatmap representing the 30 most abundant genus groups in baseline stool samples based on relative abundance; dendrograms illustrated on the x and y axes are based on Bray-Curtis distances and represent which genera (rows) or samples (columns) cluster together based on similarity in their community composition.

**FIGURE 2:**
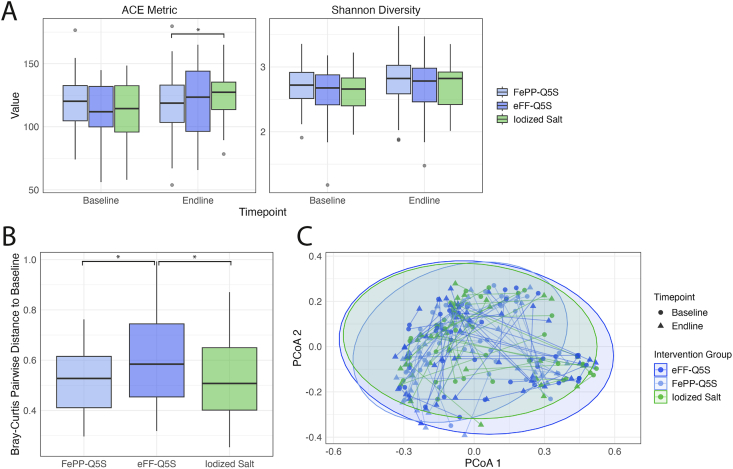
Impact of quintuply-fortified salt [QFS; QFS with iron in the form of encapsulated ferrous fumarate (eFF-Q5S) and QFS with iron in the form of ferric pyrophosphate plus EDTA (FePP-Q5S)] on the fecal microbiome in terms of alpha and beta diversity, compared with iodized salt control. (A) Effect of QFS on measures of alpha diversity [abundance-based estimator (ACE) metric and Shannon diversity] measured at baseline and endline (12 mo). Intervention effects were measured using linear mixed models evaluated using a (intervention group∗endline) interaction term in the fixed effects portion of the model. A random effect of participant ID was also included. Models were adjusted for age and additional covariates (ever breastfed, assets index, baseline hemoglobin, length-for-age Z-score, weight-for-age Z-score, baseline alpha-1 acid glycoprotein, and baseline inflammation-adjusted ferritin). (B) Boxplot of Bray-Curtis pairwise distances to baseline to identify structural changes within individuals from baseline to endline. Intervention effects were measured using linear regression models adjusting for age and additional covariates (sex, ever breastfed, baseline hemoglobin, Household Food Insecurity Access Score (HFIAS), length-for-age Z-score, weight-for-age Z-score, baseline AGP). (C) A principal coordinate (PCoA) plot of Bray-Curtis distances at baseline and endline. Differences between intervention groups were identified using permutational multivariate analysis of variance (PERMANOVA) testing on the Bray-Curtis matrix using a (intervention group∗endline) interaction term, adjusting for age and additional covariates ((sex, ever breastfed, baseline hemoglobin, HFIAS, length-for-age Z-score, weight-for-age Z-score, baseline AGP), with 999 permutations to assess statistical significance. AGP, alpha-1-glycoprotein; HFIAS,.

At baseline, *Prevotella*, *Bifidobacterium*, and *Bacteroides* were among the most abundant genera among children (Figure 1, Supplemental Table 6). There were no differences in Shannon diversity between groups at baseline. Shannon diversity also did not differ according to group at the end of the 12-mo intervention period (group-by-time interaction, *P* > 0.05) (Figure 2A). Similarly, there were no differences in ACE index according across groups at baseline. However, at 12 mo, the iodized salt group had a greater increase in diversity as measured by ACE index compared with the eFePP-QFS group (group-by-time interaction, *P* = 0.03). Changes in pairwise Bray-Curtis distances between baseline and 12 mo differed significantly between iodized salt compared with eFF-QFS groups (*P* = 0.02), and eFePP-QFS compared with eFF-QFS groups (*P* = 0.01) (Figure 2B). Visual inspection with PCoA plots and PERMANOVA testing showed that Bray-Curtis dissimilarity did not differ according to group (group-by-time interaction, overall *P* = 0.93; iodized salt compared with eFF-QFS, *P* = 0.95; iodized salt compared with eFePP-QFS, *P* = 0.50; eFF-QFS compared with eFePP-QFS, *P* = 0.95) (Figure 2C).

**FIGURE 3:**
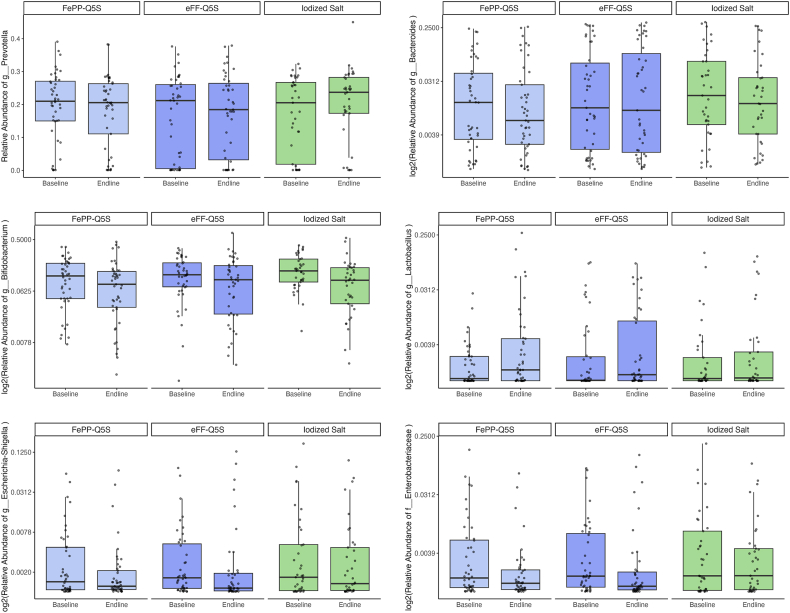
Box plots of abundance of bacterial genus groups (*Bifidobacterium*, *Lactobacillus*, *Prevotella*, *Bacteroides*, and *Escherichia-Shigella*) and the bacterial family *Enterobacteriaceae* among all 3 intervention groups [iodized salt control, quintuply-fortified salt (QFS) with iron in the form of encapsulated ferrous fumarate (eFF-Q5S), and QFS with iron in the form of ferric pyrophosphate plus EDTA (FePP-Q5S)]. Intervention effects on abundance were evaluated using zero-inflated negative binomial mixed models. The intervention effect was quantified using a (intervention group∗endline) interaction term in the fixed-effects portion of the model. Total operational taxonomic unit counts were included as an offset term to account for varying total counts in the fixed effects portion of the model. Participant ID was included as a random effect. Models were adjusted for age and additional covariates: *Prevotella* model adjusted for ever breastfed and assets index, *Lactobacillus* model adjusted for ever breastfed, baseline hemoglobin, length-for-age Z-score, weight-for-age Z-score, baseline alpha-1 acid glycoprotein (AGP), and baseline inflammation-adjusted ferritin, *Bacteroides* model adjusted for ever breastfed, Household Food Insecurity Access Score, assets index, length-for-age Z-score, weight-for-age Z-score, baseline AGP, baseline C-reactive protein, and baseline inflammation-adjusted ferritin, *Escherichia-Shigella* model adjusted for ever breastfed, baseline hemoglobin, length-for-age Z-score, baseline AGP, baseline inflammation-adjusted ferritin, Enterobacteriaceae model adjusted for ever breastfed, baseline hemoglobin, length-for-age Z-score, baseline hemoglobin and baseline inflammation-adjusted ferritin. *P* values were corrected for false-discovery rate using Benjamini-Hochberg correction.

At baseline and after 12 mo, there were no differences in the relative abundance of *Prevotella*, *Bifidobacteria*, *Lactobacillus***,** *Bacteroides*, *Escherichia-Shigella*, nor *Enterobacteriaceae* between QFS and iodized salt groups (group-by-time interaction, *q* value > 0.05) (Figure 3). Differential abundance testing via ANCOMBC identified no genus or family groups that were differentially abundant between groups (group-by-time interaction, *q* value > 0.05).

## Discussion

To our knowledge, this study is one of the first to evaluate the effects of LSFF in the form of multiply-fortified salt on gut microbiome outcomes among children 1–5 y of age. At baseline, children’s gut microbiomes closely matched findings from other studies in Northern India [[36], [37], [38], [39]]. We observed a dominance of *Prevotella*, which is common in populations consuming a predominately lacto-vegetarian dietary pattern high in carbohydrate, dietary fiber, and fermented dairy products [38,40]. Our analysis revealed some groupwise differences in alpha and beta diversity metrics. However, these differences did not consistently suggest a detrimental impact of iron fortification on gut microbial diversity or composition, despite concerns about potential adverse effects of iron on the microbiome.

Some randomized controlled trials among young children in Africa and South Asia have demonstrated negative effects of iron interventions, such as the provision of iron-containing MNPs, on both clinical and microbiome outcomes, including diarrhea, increased intestinal inflammation, decreased microbial diversity, and increased enteropathogen burden [[10], [11], [12], [13], [14],^41^]. In our study, we were specifically interested in whether the level of iron provided in QFS (1.3 mg/g of salt) would elicit any similar adverse changes to the overall composition of the microbiome, as well as abundance of potential pathogens, among children.

Across the study period, average salt utilization among all children in the microbiome subgroup was estimated to be 3.5 g/child equivalent/d and did not differ across study groups. At this level of utilization, the approximate amount of iron in the study salt was nearly 4.6 mg of iron per day. At endline, the iodized salt group showed a moderate increase in ACE index (∼14 ACE units) relative to eFePP-QFS; however, no significant difference in Shannon diversity was observed between these 2 groups. The Shannon diversity index accounts for both richness and evenness of taxa [42,43], whereas the ACE index is more sensitive to low abundance taxa [44,45]. The stability of Shannon diversity over time suggests that the overall composition and dominant taxa remained similar across intervention groups, and the shift between eFePP-QFS and the iodized salt group may have occurred among rare taxa. Furthermore, the eFF-QFS group did not differ from the iodized salt group in terms of the ACE index, suggesting the eFePP formulation may interact with the microbiome differently than eFF-QFS. However, differential abundance testing of all genera and families did not identify significant differences in abundance of any specific bacterial taxa between the iodized salt and eFePP-Q5S group, implying no harmful or pathogenic shift.

Linear regression models showed that the eFF-QFS group experienced a significantly greater change in pairwise Bray-Curtis dissimilarity from baseline to endline compared with both the iodized salt and eFePP-QFS groups. This suggests a somewhat larger compositional change over time among the children in the eFF-QFS group, as this metric examines the difference in Bray-Curtis distance from baseline to endline within individual children. However, when Bray-Curtis distance was compared between children using PERMAVOA testing on the Bray-Curtis matrix, there were no statistical differences between groups. In addition, there were no differences in alpha diversity in between the eFF-Q5S group and the other 2 groups. Thus, this effect may also be attributable to unmeasured variables that were not accounted for, such as dietary changes and current breastfeeding status, rather than the intervention.

Despite some studies demonstrating that oral iron doses >12.5 mg/d can reduce beneficial bacteria like *Lactobacillus* and promote potential pathogens like *Escherichia-Shigella* [6,[10], [11], [12]], we observed no decreases in *Lactobacillus* and *Bifidobacterium*, nor increases in *Escherichia-Shigella* or bacteria belonging to the *Enterobacteriaceae* family in any study group. This finding suggests that the doses likely delivered by QFS did not adversely affect dominant bacterial groups or increase the abundance of known enteric pathogens among children.

Adverse effects of iron interventions can depend on baseline iron status. Studies in young children indicate that providing excess iron to those who are iron-replete may lead to growth deficits [[46], [47], [48], [49]] and diarrhea [13,49,50], potentially due to iron-dependent pathogens proliferating and altering the microbiome [51]. In contrast, negative outcomes may be less pronounced in children who are iron-deficient because in the absence of inflammation, iron absorption is greater [51]. In our study, over 58% of participants across all groups were iron-deficient at baseline. Furthermore, QFS also contained additional nutrients beyond iron, including zinc, vitamin B12, and folic acid, and we observed significant improvements in parameters of B12 and folate status among all children enrolled in the main trial (results not shown here). Micronutrient deficiencies beyond iron deficiency are associated with adverse microbiome outcomes [52,53]; thus, addressing multiple deficiencies through QFS may have provided synergistic benefits.

Environmental factors, particularly hygiene and sanitation, strongly influence microbiome composition and predict adverse microbiome outcomes. In settings with poor sanitation, higher exposure to opportunistic pathogens, combined with unabsorbed dietary iron, can promote pathogen overgrowth [17,51]. Moreover, environmental enteric dysfunction, which is prevalent where sanitary conditions are suboptimal, promotes bacterial translocation into systemic circulation, driving inflammation and hepcidin release that downregulates intestinal iron absorption. However, improved sanitation reduces baseline pathogen burden and can prevent enteropathy, thereby reducing risk of pathogen proliferation exacerbated by increased iron intake. For instance, a randomized trial of iron supplements (50 mg/d, 4 d/wk) in South African children with access to an improved water supply and better hygiene found no significant effects on the microbiome or gut inflammation (fecal calprotectin) [54]. By comparison, a study of children 6–14 y in Côte d’Ivoire who had high systemic inflammation and hookworm infections saw declines in both *Bifidobacteria* and *Lactobacillus* and elevated enteric inflammation after consuming iron-fortified (20 mg) biscuits 4 times/wk [12]. Thus, interventions that deliver a lower effective daily iron dose, such as LSFF, may have less negative impact with respect to microbiome outcomes in LMIC settings where hygiene is suboptimal.

We note that in our study, children lived in relatively hygienic households, as all children lived in households with piped water and flush toilets, reported no diarrhea at sample collection, and had minimal diarrhea throughout the intervention. Potentially pathogenic genera like *Vibrio*, *Clostridium*, and *Campylobacter* were rare or undetected, indicating a low baseline pathogen burden. Although *Escherichia–Shigella* was present at low abundance in 89% of samples (Supplemental Table 3), abundance did not increase in any intervention group. We did note a relatively high prevalence of inflammation among children at baseline, especially reflected in elevated levels of AGP. We accounted for this by adjusting for inflammatory markers at baseline (AGP and CRP) in our statistical models, and we found that adjustment for inflammation did not change our results or interpretation.

Strengths of this study include its double-blind, randomized, controlled design and the use of baseline-adjusted, zero-inflated negative binomial mixed models, allowing a robust assessment of longitudinal changes in taxa while accounting for inter-individual variability. We further adjusted for baseline inflammation and micronutrient deficiencies; factors known to influence the microbiome [53]. To detect shifts in low-abundance taxa, we conducted differential abundance analysis using ANCOMBC, which appropriately handles the compositional nature of microbiome data [35]. However, the study also has limitations. We relied on 16S rRNA gene sequencing, which restricts taxonomic resolution to the genus level and prevents species- or strain-level inferences. We did not include metagenomic or metatranscriptomic analyses, limiting our ability to identify functional changes in microbial communities. Only 2 collection time points (baseline and 12-mo endline) were used, reducing the opportunity to capture more nuanced temporal dynamics or acute shifts. Our study was also constrained by the sample size; a larger sample would have provided greater statistical power to detect more subtle shifts in the microbiome. In addition, the children in our sample represented a large range of ages between 1 and 5 y. This variability may have affected our ability to determine whether changes were due to the intervention itself, or due to age-related changes in the developing microbiome. However, age was not statistically different across intervention groups, and the median age in our sample was over 3 y, suggesting that most children were beyond the more rapid early-life shifts, likely having relatively stable microbiomes [55]. In addition, all statistical models were adjusted for age and breastfeeding to account for age-related variation. Although we measured systemic inflammatory markers, we did not collect fecal markers of intestinal inflammation or enteric dysfunction (e.g., fecal calprotectin), limiting insights into gut inflammatory processes. In addition, the fortification levels of the multiply-fortified salt were based on women’s intake data and did not directly account for children’s intake of discretionary salt. Lastly, the study was conducted in a single region in Punjab, India, with relatively hygienic conditions, and among children with relatively poor iron status, which may limit generalizability to populations with different environmental exposures or baseline pathogen burden, as well as populations with better iron status.

To our knowledge, this is the first study to examine microbiome changes in response to LSFF in the form of multiply-fortified salt among children 1–5 y of age. We noted minimal and inconsistent shifts in certain microbial diversity metrics, and no major changes in abundance of taxa. Importantly, we did not observe increases in *Escherichia-Shigella* or *Enterobacteriaceae*, nor decreases in abundance of *Lactobacillus* or *Bifidobacterium*, suggesting that QFS did not induce substantial or adverse microbiome alterations related to increased abundance of potentially pathogenic bacteria. Future studies should confirm these findings by examining children in regions with higher enteropathogen burdens, utilizing a larger sample size, and incorporating additional assessments (functional metagenomics, strain-level analyses, or more frequent sampling) to capture subtle temporal changes and confirm the safety and efficacy of LSFF interventions across diverse settings.

## Author contributions

The authors’ responsibilities were as follows – LT, YEG, NFK, AZ, MD, RD, CMM: designed the research; LT, YEG, MJ, BLS, GKB, JW, JML: conducted the research; LT, CDA: analyzed the data; LT: wrote the paper; LT: had primary responsibility for final content; and all authors: read and approved the final manuscript.

## Data availability

Data described in the manuscript, code book, and analytic code will be made available upon request. Sequencing reads have been deposited in the Sequence Read Archive (PRJNA1208124).

## Funding

This research was funded by the Bill & Melinda Gates Foundation (INV-002945) and the Waterloo Foundation (2757-5391, CA-0237773)

## Conflict of interest

The authors report no conflicts of interest.
