# Supplementary material for: The Effect of Quintuply-Fortified Salt on the Gut Microbiome of Young Children 1–5 y of Age in Punjab, India; A Substudy of a Randomized, Community-Based Trial
Source: Curr Dev Nutr. 2025 Oct 23;9(11):107580. doi: 10.1016/j.cdnut.2025.107580 (PMC12664039; doi:10.1016/j.cdnut.2025.107580)
Supplement: Multimedia component 1 [file mmc1.docx]

**Online Supplement:** The Effect of Quintuply-Fortified Salt on the Gut Microbiome of Young Children 1-5 Years of Age in Punjab, India; A Substudy of a Randomized, Community-Based Trial

**Lauren Thompson et al.**

Children enrolled into MFS trial (n = 470)

Children with paired samples available for 16S sequencing

(n = 43)

Children randomized to iodized salt group

(n = 149)

Children randomized to eFePP-QFS group

(n = 160)

Children randomized to eFF-QFS group

(n = 161)

Children randomly selected to microbiome subgroup

(n = 54)

Children randomly selected to microbiome subgroup (n = 61)

nn

Children randomly selected to microbiome subgroup (n = 56)

Children with paired samples available for 16S sequencing

(n = 37)

Children with paired samples available for 16S sequencing

(n = 45)

**Excluded (n = 17)**

- No longer interested (n = 2)
- Did not like salt (n = 3)
- Moved from study area (n = 1)
- Household no longer eligible (n = 2)
- Refused endline stool sample (n = 8)
- Final classified read depth below 10,000 reads (n = 1)

**Excluded (n = 16)**

- No longer interested (n = 4)
- Afraid of blood draws (n = 1)
- Did not like salt (n = 3)
- Moved from study area (n = 1)
- Household no longer eligible (n = 2)
- Refused endline stool sample (n = 3)
- Final classified read depth below 10,000 reads (n = 2)

**Excluded (n = 13)**

- No longer interested (n = 3)
- Household no longer eligible (n = 7)
- Refused endline stool sample (n = 2)
- Final classified read depth below 10,000 reads (n = 1)

**Supplemental Figure 1:** Flow diagram depicting the allocation of children 1-5 years of age to the different arms of the Multiply Fortified Salt (MFS) trial. A subset of children (n = 171) were randomized to the microbiome subgroup (n = 55 in the iodized salt group, n = 64 in the QFS with iron in the form of encapsulated ferrous fumarate (eFF-QFS); group, and n = 57 in the QFS with iron in the form of ferric pyrophosphate plus ethylenediaminetetraacetic acid (EDTA) (FePP-QFS) group). Samples were excluded from the analysis if children did not provide both a baseline and stool sample or did not meet eligibility criteria to continue in the study (iodized salt n = 37, FePP-Q5S n = 45, eFF-QFS n = 43).

**Supplemental Table 1:** PCR Primer Sequences

| **Primer** | **Sequence** |
| --- | --- |
| 16s rRNA Forward Primer | 5'AGAGTTTGATCCTGGCTCAG'3 |
| 16s rRNA Reverse Primer | 5'GGTTACCTTGTTACGACTT'3 |
| V3-V4 Forward Primer | CCTACGGGNGGCWGCAG |
| V3-V4 Reverse Primer | GACTACHVGGGTATCTAATCC |

**Supplemental Table 2:** Reagents used for 16S PCR

| Components | Volume (μl) |
| --- | --- |
| NEB 2X High Fidelity Master Mix | 12.5 μl |
| 16S Forward primer (10pm/ul) | 1 μl |
| 16S Reverse Primer (10pm/ul) | 1 μl |
| Template (5ng) |  |
| UV-irradiated Nuclease-free water |  |
| Total volume | 25 μl |

Abbreviations:

NEB: New England Biolabs

**Supplemental Table 3:** PCR Conditions for 16S PCR

| Temperature | Duration | Step |
| --- | --- | --- |
| 95°C | 3 min | 1 cycle |
| 95°C | 15 sec | 7 cycles |
| 48°C-54°C | 20 sec |  |
| 72°C | 1 min |  |
| 95°C | 15 sec | 35 cycles |
| 55°C | 20 sec |  |
| 72°C | 1 min |  |
| 72°C | 7 min | 1 cycle |
| 4°C | ∞ | Hold |

**Supplemental Table 4:** Reagents used for V3-V4 PCR

| Components | Volume (μl) |
| --- | --- |
| UV-irradiated Nuclease free water (NFW) | 9.5 μl |
| NEB 2X High Fidelity Master Mix | 12.5 μl |
| V3-V4 Forward Primer (10pm/ul) | 1 μl |
| V3-V4 Reverse Primer (10pm/ul) | 1 μl |
| 16S Template (1:1 Dilution) | 1 μl |
| Total volume | 25 μl |

**Supplemental Table 5:** PCR Conditions for V3-V4 PCR

| Temperature | Duration | Step |
| --- | --- | --- |
| 95°C | 3 min | 1 cycle |
| 95°C | 30 sec | 5 cycles |
| 50°C-54°C | 30 sec |  |
| 72°C | 30 sec |  |
| 95°C | 30 sec | 35 cycles |
| 55°C | 30 sec |  |
| 72°C | 30 sec |  |
| 72°C | 7 min | 1 cycle |
| 4°C | ∞ | Hold |

**Supplemental Table 6:** Median relative abundance and detection of top 35 taxa present in baseline samples

| **Taxon** | **Median Abundance (%)** | **Presence in Samples (%)** |
| --- | --- | --- |
| d__Bacteria;p__Bacteroidota;c__Bacteroidia;o__Bacteroidales;f__Prevotellaceae;g__Prevotella | 21.084% | 93.60% |
| d__Bacteria;p__Actinobacteriota;c__Actinobacteria;o__Bifidobacteriales;f__Bifidobacteriaceae;g__Bifidobacterium | 12.526% | 100.00% |
| d__Bacteria;p__Firmicutes;c__Clostridia;o__Oscillospirales;f__Ruminococcaceae;g__Faecalibacterium | 8.261% | 98.40% |
| d__Bacteria;p__Firmicutes;c__Clostridia;o__Lachnospirales;f__Lachnospiraceae;__ | 5.393% | 100.00% |
| d__Bacteria;p__Bacteroidota;c__Bacteroidia;o__Bacteroidales;f__Bacteroidaceae;g__Bacteroides | 1.364% | 100.00% |
| d__Bacteria;p__Firmicutes;c__Clostridia;o__Lachnospirales;f__Lachnospiraceae;g__Blautia | 4.085% | 100.00% |
| d__Bacteria;p__Actinobacteriota;c__Coriobacteriia;o__Coriobacteriales;f__Coriobacteriaceae;g__Collinsella | 2.251% | 93.60% |
| d__Bacteria;p__Firmicutes;c__Clostridia;o__Lachnospirales;f__Lachnospiraceae;g__Roseburia | 1.875% | 97.60% |
| d__Bacteria;p__Firmicutes;c__Negativicutes;o__Veillonellales-Selenomonadales;f__Veillonellaceae;g__Dialister | 1.646% | 84.00% |
| d__Bacteria;p__Firmicutes;c__Bacilli;o__Lactobacillales;f__Streptococcaceae;g__Streptococcus | 0.704% | 100.00% |
| d__Bacteria;p__Firmicutes;c__Bacilli;o__Erysipelotrichales;f__Erysipelatoclostridiaceae;g__Asteroleplasma | 0.001% | 52.00% |
| d__Bacteria;p__Firmicutes;c__Clostridia;o__Lachnospirales;f__Lachnospiraceae;g__Agathobacter | 0.516% | 98.40% |
| d__Bacteria;p__Firmicutes;c__Negativicutes;o__Veillonellales-Selenomonadales;f__Selenomonadaceae;g__Megamonas | 0.057% | 67.20% |
| d__Bacteria;p__Firmicutes;c__Bacilli;o__Erysipelotrichales;f__Erysipelatoclostridiaceae;g__Catenibacterium | 0.038% | 63.20% |
| d__Bacteria;p__Firmicutes;c__Clostridia;o__Lachnospirales;f__Lachnospiraceae;g__Anaerostipes | 0.698% | 97.60% |
| d__Bacteria;p__Firmicutes;c__Clostridia;o__Oscillospirales;f__Ruminococcaceae;g__Subdoligranulum | 0.943% | 92.80% |
| d__Bacteria;p__Firmicutes;c__Clostridia;o__Lachnospirales;f__Lachnospiraceae;g__Dorea | 0.880% | 96.80% |
| d__Bacteria;p__Verrucomicrobiota;c__Verrucomicrobiae;o__Verrucomicrobiales;f__Akkermansiaceae;g__Akkermansia | 0.002% | 57.60% |
| d__Bacteria;p__Firmicutes;c__Clostridia;o__Lachnospirales;f__Lachnospiraceae;g__[Eubacterium]_hallii_group | 0.767% | 92.80% |
| d__Bacteria;p__Bacteroidota;c__Bacteroidia;o__Bacteroidales;f__Tannerellaceae;g__Parabacteroides | 0.120% | 91.20% |
| d__Bacteria;p__Firmicutes;c__Bacilli;o__Erysipelotrichales;f__Erysipelotrichaceae;g__Holdemanella | 0.001% | 52.00% |
| d__Bacteria;p__Bacteroidota;c__Bacteroidia;o__Bacteroidales;f__Prevotellaceae;g__Alloprevotella | 0.000% | 49.60% |
| d__Bacteria;p__Proteobacteria;c__Gammaproteobacteria;o__Aeromonadales;f__Succinivibrionaceae;g__Succinivibrio | 0.000% | 24.80% |
| d__Bacteria;p__Proteobacteria;c__Gammaproteobacteria;o__Burkholderiales;f__Sutterellaceae;g__Sutterella | 0.485% | 95.20% |
| d__Bacteria;p__Proteobacteria;c__Gammaproteobacteria;o__Enterobacterales;f__Enterobacteriaceae;g__Escherichia-Shigella | 0.056% | 88.80% |
| d__Bacteria;p__Firmicutes;c__Clostridia;o__Lachnospirales;f__Lachnospiraceae;g__Fusicatenibacter | 0.306% | 88.80% |
| d__Bacteria;p__Firmicutes;c__Negativicutes;o__Veillonellales-Selenomonadales;f__Veillonellaceae;g__Megasphaera | 0.001% | 51.20% |
| d__Bacteria;p__Firmicutes;c__Clostridia;o__Oscillospirales;f__Oscillospiraceae;g__UCG-002 | 0.130% | 84.00% |
| d__Bacteria;p__Firmicutes;c__Clostridia;o__Lachnospirales;f__Lachnospiraceae;g__Coprococcus | 0.289% | 89.60% |
| d__Bacteria;p__Firmicutes;c__Clostridia;o__Lachnospirales;f__Lachnospiraceae;g__[Ruminococcus]_gnavus_group | 0.051% | 94.40% |
| d__Bacteria;p__Firmicutes;c__Bacilli;o__Lactobacillales;f__Lactobacillaceae;g__Lactobacillus | 0.007% | 62.40% |
| d__Bacteria;p__Firmicutes;c__Clostridia;o__Lachnospirales;f__Lachnospiraceae;g__Lachnospiraceae_NK4A136_group | 0.227% | 93.60% |
| d__Bacteria;p__Firmicutes;c__Clostridia;o__Oscillospirales;f__Ruminococcaceae;g__Ruminococcus | 0.266% | 91.20% |
| d__Bacteria;p__Firmicutes;c__Clostridia;o__Lachnospirales;f__Lachnospiraceae;g__[Ruminococcus]_torques_group | 0.129% | 96.80% |
| d__Bacteria;p__Firmicutes;c__Clostridia;o__Oscillospirales;f__[Eubacterium]_coprostanoligenes_group;g__[Eubacterium]_coprostanoligenes_group | 0.249% | 84.00% |
